# Robust approaches for model-free small-angle scattering data analysis^1^

**DOI:** 10.1107/S1600576722004356

**Published:** 2022-05-28

**Authors:** Philipp Bender, Dirk Honecker, Mathias Bersweiler, Rocio Costo, Tamara Kahmann, Frank Ludwig, Jon Leiner, Johanna K. Jochum

**Affiliations:** a Heinz Maier-Leibnitz Zentrum (MLZ), Technische Universität München, D-85748 Garching, Germany; b ISIS Neutron and Muon Facility, Rutherford Appleton Laboratory, Chilton, OX11 0QX, United Kingdom; c Physics and Materials Science Research Unit, University of Luxembourg, 162A avenue de la Faïencerie, L-1511 Luxembourg, Grand Duchy of Luxembourg; d Instituto de Ciencia de Materiales de Madrid, ICMM/CSIC, C/Sor Juana Inés de la Cruz 3, 28049 Madrid, Spain; e Institute of Electrical Measurement Science and Fundamental Electrical Engineering and Laboratory for Emerging Nanometrology (LENA), Technische Universität Braunschweig, 38106 Braunschweig, Germany

**Keywords:** small-angle scattering, correlation functions, Fourier transform, magnetic nanoparticles, modulation of intensity with zero effort, MIEZE, RESEDA

## Abstract

The small-angle neutron scattering data of nanostructured magnetic samples contain information regarding their chemical and magnetic properties. Often, the first step to access characteristic magnetic and structural length scales is a model-free investigation. However, due to measurement uncertainties and a restricted *q* range, a direct Fourier transform usually fails and results in ambiguous distributions. To circumvent these problems, different methods have been introduced to derive regularized, more stable correlation functions, with the indirect Fourier transform being the most prominent approach. Here, the indirect Fourier transform is compared with the singular value decomposition and an iterative algorithm. These approaches are used to determine the correlation function from magnetic small-angle neutron scattering data of a powder sample of iron oxide nanoparticles; it is shown that with all three methods, in principle, the same correlation function can be derived. Each method has certain advantages and disadvantages, and thus the recommendation is to combine these three approaches to obtain robust results.

## Abstract 2

Three different approaches are compared for determination of the correlation function from the small-angle neutron scattering data of a powder sample of iron oxide nanoparticles.

## 1. Introduction

Small-angle neutron scattering (SANS) probes chemical and magnetic structure on the mesoscale (∼1–500 nm) (Jeffries *et al.*, 2021 ▸), which makes SANS an ideal tool to investigate nanostructured magnetic materials such as bulk ferromagnets or magnetic nanoparticle systems (Mühlbauer *et al.*, 2019 ▸). In SANS data analysis, it is good practice to perform a Fourier transform to obtain starting parameters for the characteristic magnetic and structural length scales that are relevant for a system (Feigin & Svergun, 1987 ▸).

In the case of pure nuclear scattering the extracted correlation function corresponds to the autocorrelation function of the scattering length density profile (Li *et al.*, 2016 ▸). This is not the case for magnetic neutron scattering due to the anisotropic nature of the dipole–dipole interaction (Mettus & Michels, 2015 ▸). However, the derived correlation functions still contain important information that reflects the real-space magnetization over the mesoscale (Bender *et al.*, 2021 ▸). Thus, the Fourier transform of reciprocal SANS data is an easy and straightforward approach to obtain model-independent information regarding the chemical and magnetic nanostructure of the sample.

Real experimental data usually have measurement uncertainties and a restricted *q* range, which can lead to ambiguous correlation functions when performing a direct Fourier transform of the data. To circumvent these issues the indirect Fourier transform (IFT) was introduced in the 1970s (Glatter, 1977 ▸). In this case, the correlation function is essentially obtained by a fit of the experimental data using a Tikhonov regularization to force smooth distributions. To find the optimal value for the smoothing degree a Bayesian analysis can be applied (Hansen, 2000 ▸). This approach allows for the inclusion of the maximum size of the scattering, which defines the size range for which the correlation function is computed, as fit parameter (Hansen, 2012 ▸). Such a model-free analysis of scattering data has proven to be a powerful approach to study several categories of materials, *e.g.* polymer solutions (Ham­mouda, 2010 ▸), protein conformation (Sanchez-Fernandez *et al.*, 2017 ▸) and the structure of colloidal particles (Fritz *et al.*, 2000 ▸).

The IFT can further be used to derive the 2D correlation functions from the complete scattering pattern (Fritz-Popovski, 2013 ▸). However, this approach is computationally intensive for large data sets. Therefore, two faster numerical approaches were recently introduced to determine the 2D correlation functions from scattering patterns, namely the singular value decomposition (SVD) (Bender *et al.*, 2019 ▸) and the iterative Kaczmarz algorithm (KA) (Bender *et al.*, 2021 ▸).

Here, we analyze 1D scattering data and apply all three model-free approaches, namely the IFT, SVD and KA. We use these methods to determine the correlation functions of a powder sample of magnetic iron oxide nanoparticles from the magnetic-field-dependent SANS intensities measured at the multipurpose neutron spin-echo spectrometer RESEDA in modulation of intensity with zero effort (MIEZE)-SANS mode (Franz, Säubert *et al.*, 2019 ▸; Franz, Soltwedel *et al.*, 2019 ▸; Jochum *et al.*, 2019 ▸).

## 2. Methods

### 2.1. Pre-characterization of the sample

**Figure 1:**
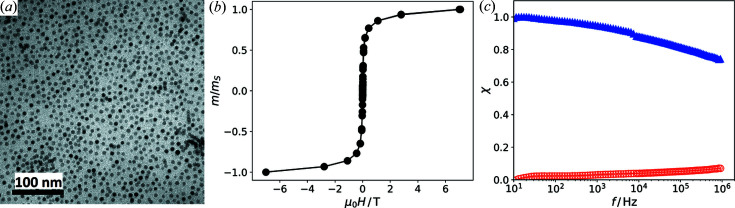
(*a*) TEM image of the iron oxide nanoparticles. (*b*) DCM measurement of the powder sample of nanoparticles normalized to the magnetic moment 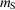 measured at saturation. (*c*) Normalized real (full blue triangles) and imaginary parts (open red circles) of the ACS measurement.

The sample was a dense powder of ∼200 mg freeze-dried iron oxide nanoparticles. The spherical single-crystalline particles were synthesized by thermal decomposition and had an average diameter of around 10 nm with a very narrow size distribution (σ < 0.1). Fig. 1 ▸(*a*) shows a transmission electron microscopy (TEM) image of the nanoparticles.

The macroscopic magnetic properties of the powder at 300 K were determined by quasistatic direct-current magnetometry (DCM) and alternating-current susceptibility (ACS) [see Figs. 1 ▸(*b*) and 1 ▸(*c*)]. The DCM measurement, measured from 6 T → −6 T → 6 T, exhibits a Langevin-type magnetization behavior with vanishing coercivity and remanence, indicating a superparamagnetic behavior of the sample. This is verified by the ACS measurement whose imaginary part is close to zero at low frequencies (Ludwig *et al.*, 2017 ▸). This means that the magnetic moments of the nanoparticles can freely follow the external magnetic field via Néel-type relaxation. With increasing frequency the imaginary part slightly increases and the real part decreases accordingly. However, within the accessible frequency range (*i.e.* 10–10^6^ Hz) no relaxation peak in the imaginary part is observed, which indicates that the characteristic relaxation times of the particles are significantly below τ = 1/ω = 1/(2π*f*) < 1.6 × 10^−7^ s.

### 2.2. Magnetic SANS measurements

**Figure 2:**
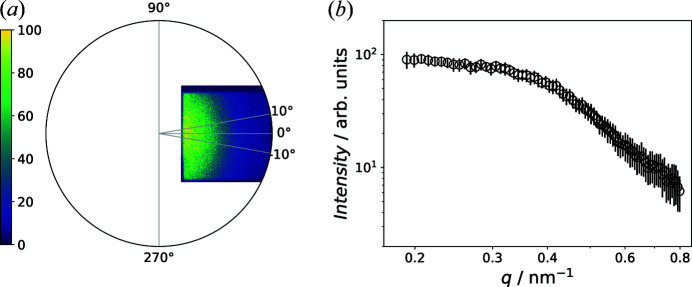
(*a*) 2D SANS pattern of the powder sample of iron oxide nanoparticles measured at zero field with the instrument RESEDA. The 2D detector was positioned off-center of the neutron beam. (*b*) Sector average along the horizontal (*i.e.* field) direction (±10°).

For the SANS measurements the particle powder was placed into a quartz glass cuvette with an optical path length of 1 mm. The scattering patterns were measured with the neutron spin-echo spectrometer RESEDA using the MIEZE-SANS arm (Franz, Soltwedel *et al.*, 2019 ▸). The 2D detector was positioned off-center of the direct neutron beam as shown in Fig. 2 ▸. The neutron wavelength was 6 Å with a wavelength spread Δλ/λ of 11.7%. The experimental setup included a magnet with the field applied in the horizontal plane. From the scattering patterns we determined the sector average in the horizontal direction, *i.e.* along the magnetic field direction, with a sector width of ±10°. In the following, we use three different approaches to derive the underlying correlation functions from the 1D sector averages.

## 3. Data analysis

### 3.1. Introduction of the three model-free approaches

From the 1D scattering intensity shown in Fig. 2 ▸, the underlying correlation function 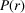 was determined by three different approaches. First the IFT was applied. In this case the *N*-dimensional vector 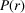 is determined by minimizing the functional 

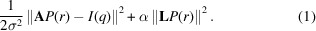

Here, 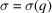 is the standard deviation of each data point and 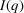 is the measured scattering intensity with *M* data points. The matrix **A** in equation (1) is the 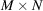 data transfer matrix with 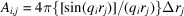 . The matrix **L** is an 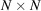 regularization matrix, which is multiplied with the regularization parameter α. To penalize oscillations within the extracted distributions, the non-singular approximation of the discrete second-order derivative operator can be used for **L**: 

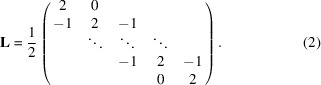

For numerical computation, equation (1) is inconvenient and the least-squares solution of 

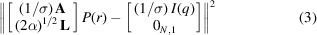

is determined, where 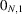 is a zero vector of length *N*. To perform the IFT the size range 0–*D*_max_ of 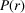 has to be defined *a priori*. However, 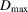 can be included as an additional fit parameter. Here, we performed the IFT for a total of 251 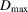 values (between 10 and 30 nm). Furthermore, we varied α over six orders of magnitude (from 10^0^ to 10^5^) and determined for each set of 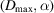 the evidence according to Hansen (2000 ▸) and as described in detail by Bender *et al.* (2017 ▸). The 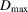 value for which the highest evidence was found was then used for the SVD and the KA as well.

With the SVD the data transfer matrix **A** is decomposed according to 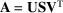 , where **U** and **V** are orthogonal 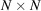 and 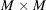 matrices, respectively, and **S** is an 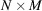 matrix whose main diagonal elements are the singular values 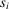 . The correlation function 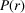 is then calculated using 

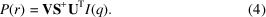

The diagonal of 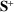 contains the reciprocal values 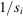 , and all off-diagonal entries of 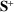 are zero. In the case of ill-posed problems, many of the singular values will be very small which amplifies measurement uncertainties. Thus, using all singular values usually results in large unphysical oscillations in the derived distributions. The singular values of *S* are usually given in descending order, and thus a smoothing can be accomplished by reducing the number of singular values 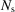 which are considered for the reconstruction (Berkov *et al.*, 2000 ▸).

Using the KA, 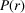 is calculated by updating the elements 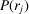 after each iteration according to

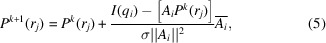

where 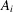 is the *i*th row of the matrix 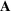 , 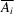 is its transpose, *k* is the iteration number, and one iteration contains a sweep over all rows *i*. Here we shuffle randomly through all rows 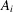 and normalize the residuals [*i.e.* 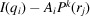 ] to 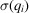 , similar to a weighted least-squares fit.

### 3.2. Results of the model-free data analysis

**Figure 3:**
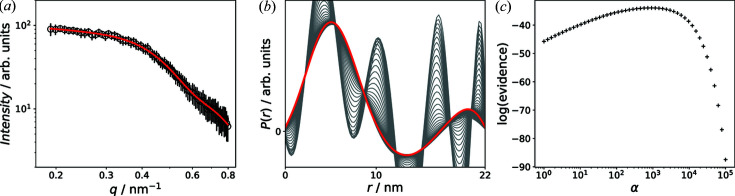
(*a*) Fit of the 1D sector average at zero field by IFT. (*b*) Correlation functions 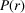 determined for all α values at 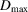 = 22 nm. The 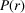 with highest evidence is marked red. (*c*) The evidences computed for all α values.

Fig. 3 ▸ shows the results for the IFT. The highest evidence was found for 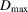 = 22 nm. The fit of the data is shown in Fig. 3 ▸(*a*). In Fig. 3 ▸(*b*) the correlation functions for all α values are plotted and the corresponding evidences are shown in Fig. 3 ▸(*c*). The 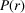 with the highest evidence is highlighted in red. The correlation function exhibits one oscillation with the first zero crossing at around 10 nm. This value agrees well with the physical particle size. The oscillations can be attributed to a structure factor due to inter-particle interference (Fritz-Popovski *et al.*, 2011 ▸), which leads to a shift of the zero crossing towards smaller sizes and concerns nuclear and magnetic contributions as discussed at the end of this section.

**Figure 4:**
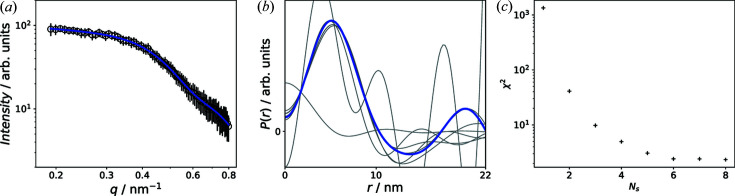
(*a*) Fit of the 1D sector average at zero field by the SVD. (*b*) Correlation functions 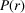 determined for a varying number of singular values ( 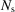 = 1–8). The 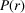 reconstructed with 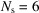 is marked in blue. (*c*) The total weighted error 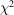 as a function of 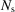 .

Fig. 4 ▸ shows the results for the SVD. As can be seen, by increasing the number of singular values that are considered for the reconstruction of 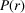 to 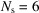 , the total weighted error 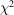 decreases [Fig. 4 ▸(*c*)] before leveling out. But for 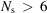 , large unphysical oscillations are obtained in the resultant correlation functions 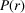 , as shown in Fig. 4 ▸(*b*). The 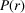 determined for 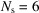 is highlighted in blue and the corresponding fit of 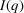 is plotted in Fig. 4 ▸(*a*).

**Figure 5:**
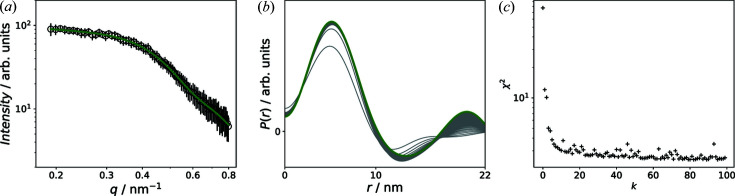
(*a*) Fit of the 1D sector average at zero field by the KA. (*b*) Correlation functions 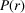 determined after all 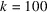 iteration steps. The 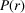 reconstructed after 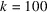 is plotted in green. (*c*) The total weighted error 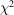 computed after each iteration step *k*.

Fig. 5 ▸ shows the results for the KA. As plotted in Fig. 5 ▸(*c*), by increasing the iterations of the KA the total error 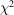 tends to decrease until approaching 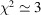 , similar to the SVD. The correlation function obtained after 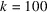 iteration steps is plotted in green in Fig. 5 ▸(*b*) and the corresponding fit is shown in Fig. 5 ▸(*a*).

**Figure 6:**
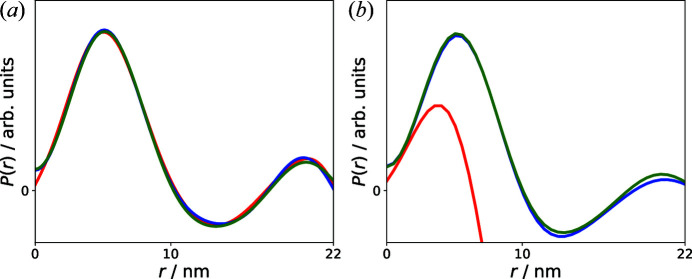
(*a*) Comparison of the correlation functions determined by the IFT [red, from Fig. 3 ▸(*b*)], SVD [blue, from Fig. 4 ▸(*b*)] and KA [green, from Fig. 5 ▸(*b*)] at zero field. 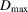 = 22 nm was determined by the IFT. (*b*) Comparison of the correlation functions determined by the IFT (red), SVD (blue) and KA (green) when 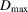 is fixed to 50 nm.

Fig. 6 ▸(*a*) shows the correlation functions determined by the IFT, the SVD and the KA. All three curves are basically identical. However, when 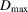 is not determined by the IFT but randomly chosen, this is not the case anymore. Fig. 6 ▸(*b*) shows as an example what happens when 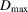 is fixed to 50 nm. The correlation functions determined by the SVD and KA are basically the same as before, whereas the IFT results in a completely different one. This inability to automatically determine the appropriate maximum accessible sizes for the system and the need for a sophisticated guess by an informed user are significant issues for most of the common available tools to estimate 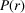 .

To better understand the physical meaning of 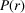 , we will now analyze the field-dependent data.

**Figure 7:**
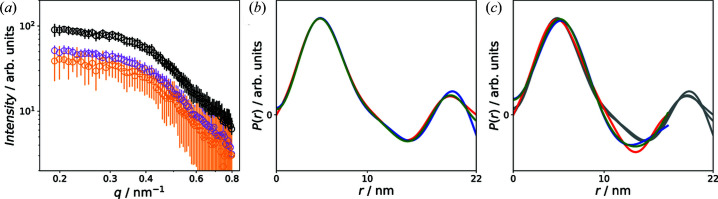
(*a*) 1D sector averages along the field direction at zero field [black, same as in Fig. 2 ▸(*b*)] and at 3 T (magenta) and the difference between the two (orange). (*b*) Comparison of the correlation functions determined by the IFT (red), SVD (blue) and KA (green) at 3 T. (*c*) Comparison of the correlation functions determined by the IFT (red), SVD (blue) and KA (green) from the difference between the zero field and the 3 T measurement. The gray curves are the distributions from panel (*b*).

Fig. 7 ▸(*a*) shows the sector averages at 3 and 0 T [as shown in Fig. 2 ▸(*b*)], as well as the difference between the two. The sectors are parallel to the applied field and thus the scattering intensity 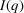 at 3 T is dominated by the nuclear scattering intensity as the sample is nearly completely magnetically saturated at this field [see Fig. 1 ▸(*b*)]. Therefore, the correlation functions derived from 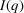 are essentially the autocorrelation function of the nuclear scattering profile, and thus the oscillations in 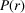 shown in Fig. 7 ▸(*b*) can be attributed to the nuclear structure factor (Weyerich *et al.*, 1999 ▸). Negative values of 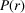 are associated with distances that connect particle volumes with a scattering length density below the average. The difference between the scattering intensities measured at zero field and 3 T, on the other hand, is of purely magnetic origin (Mühlbauer *et al.*, 2019 ▸). Thus, the derived correlation function 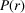 contains information regarding the moment correlations between neighboring particles at zero field. The observed negative values at *r* > 10 nm indicate antiferromagnetic-like moment correlations similar to what was observed by Bender *et al.* (2018 ▸). A comparison of the magnetic correlation functions with the distributions from Fig. 7 ▸(*a*) shows pronounced differences in the range 10–15 nm. This demonstrates that the nuclear and magnetic structure factors are not the same (Honecker *et al.*, 2020 ▸). Furthermore, the magnetic correlation functions have no second positive peak in the range 17–22 nm, which indicates that magnetic order only exists between nearest neighbors. For larger distance, no correlation exists due to thermal fluctuations of the particle moments. Regarding the comparison of the three approaches for the determination of 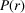 we reiterate that all three approaches result in very similar correlation functions [Figs. 7 ▸(*b*) and 7 ▸(*c*)].

## 4. Discussion and summary

Here, we have analyzed the magnetic-field-dependent SANS pattern of a powder sample of 10 nm iron oxide nanoparticles measured with the neutron resonant spin-echo spectrometer RESEDA. Our analysis of the 1D sector averages shows that the IFT, SVD and KA all result in identical correlation functions 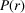 . IFT is the standard approach for such problems and well established. In comparison with the IFT, both SVD and KA are less sensitive to the size range chosen for the reconstruction of 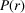 . This means that, for the IFT, the 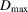 value has to be included as a fit parameter, but this is not the case for the SVD and KA, which also significantly reduces the computational costs. In general, an advantage of the SVD and KA is the faster computation times. While this is negligible when handling 1D data, it is a huge advantage for 2D data analysis as shown *e.g.* by Bender *et al.* (2019 ▸, 2021 ▸). Regarding an automated data analysis, the KA in particular has great potential as also discussed in the context of other measurement techniques (Karpavičius *et al.*, 2021 ▸). This approach could also be applied when simultaneously analyzing data sets of complementary characterization techniques. Such a global fit could be pursued for dilute spherical nanoparticle systems, *e.g.* to determine the functional form of the size distribution or the magnetic volume without skewing the data to a given, potentially false, distribution (*e.g.* log-normal) form.

Regarding small-angle scattering data analysis, we recommend applying the SVD and KA approaches in addition to the IFT, as a consistent result with all three methods significantly strengthens the confidence in the obtained correlation functions. The corresponding Python scripts can be found at https://github.com/PBenderLux/Data-analysis and are free to use. Furthermore, the SVD and KA will be implemented in the open-source *SasView* software package to provide easy access for data analysis.
